# Cytokine profiles of *Necator americanus* and *Plasmodium falciparum* co-infected patients in rural Ghana

**DOI:** 10.1016/j.cytox.2019.100014

**Published:** 2019-10-04

**Authors:** Benjamin Amoani, Bright Adu, Margaret T. Frempong, Tracy Sarkodie-Addo, Samuel Victor Nuvor, Emmanuel Kwasi Abu, Lisa M. Harrison, Michael Cappello, Ben Gyan, Michael D. Wilson

**Affiliations:** a Department of Biomedical Science, College of Health Sciences, University of Cape Coast, Cape Coast, Ghana; b Department of Immunology, Noguchi Memorial Institute for Medical Research, College of Health Sciences, University of Ghana, Legon, Ghana; c Molecular Medicine Department, School of Medical Sciences, Kwame Nkrumah University of Science and Technology, Ghana; d Department of Microbiology, College of Health Sciences, University of Cape Coast, Cape Coast, Ghana; e Department of Optometry, College of Health Sciences, University of Cape Coast, Cape Coast, Ghana; f Partnerships for Global Health, Department of Pediatrics, Yale School of Medicine, Yale University, New Haven, CT, USA; g Parasitology Department, Noguchi Memorial Institute for Medical Research, College of Health Sciences, University of Ghana, Legon, Ghana

**Keywords:** *Necator americanus*, *Plasmodium falciparum*, Hookworm, Cytokine, Infection intensity, Albendazole treatment, CCL11, Eotaxin, EPG, Egg per Gram, STHs, Soil Transmitted Helminths

## Abstract

**Background:**

*Necator americanus* (hookworm) and *Plasmodium falciparum* co-infections are common in endemic communities in rural Ghana. Human immune responses to *P. falciparum* and hookworm are complex, and the dynamics of cytokine levels and effector mediators are poorly understood. This study aimed to determine the effect of hookworm and *P. falciparum* co-infection on parasite intensities and cytokine profiles in individuals before and after deworming drug treatment.

**Methods:**

In this cross-sectional study conducted in the Kintampo North Municipality of Ghana blood and stool samples were analyzed from 984 participants (aged 4–88 years). Stool samples were collected at baseline from all participants and examined for the presence of hookworm using the Kato-Katz method. Blood and stool samples were analysed again two weeks after albendazole treatment of hookworm infected individuals. Malaria parasitaemia was estimated by light microscopy and *P. falciparum*-specific 18S rRNA gene PCR method used for species identification. Serum levels of circulating cytokines interleukins -5, -10 (IL-5, IL-10), tumor necrosis factor [TNF]-α, and eotaxin [CCL11] were determined using ELISA based methods.

**Results:**

Malaria parasitaemia was significantly reduced in hookworm and *P. falciparum* co-infected individuals (p = 0.0018) while hookworm intensity was similar between groups. IL-10 level was significantly higher in the co-infected individuals (39.9 ± 12.2 pg/ml) compared to the single infected or the uninfected group (10.7 ± 7.6 mg/ml). IL-5 level was higher in the hookworm only infected individual. TNF-α levels were higher in all infected groups compared to the uninfected controls. CCL11 levels were significantly higher in subjects infected with hookworm only or co-infected with hookworm and *P. falciparum.* There was a significantly negative correlation (r_s_ = −0.39, p = 0.021) between hookworm eggs per gram of stool and CCL11 levels in the group mono-infected with hookworm which was not affected by treatment. Treatment with albendazole led to a significant reduction of TNF-α (p = 0.041), IL-5 (p = 0.01) and IL-10 (p = 0.001) levels.

**Conclusion:**

This study shows that in the absence of other helminths, co-infection of hookworm with *P. falciparum* may modulate blood parasitemia levels and cytokine responses. Data also show that deworming drug treatment alters these cytokine profiles in hookworm infected subjects. Future studies to elucidate the potential mechanisms underlying these observations should include an assessment of parasite specific cellular responses.

## Highlights

• Co-infection of hookworm with *P. falciparum* modulates blood parasitemia levels.
• Cytokine levels were higher in the parasite infected individuals.
• Serum eotaxin level correlate negatively with hookworm intensity.
• Deworming drug treatment alters cytokine profiles in hookworm infected subjects.

## 1 Introduction

Hookworm and malaria co-infections are common among individuals in malaria endemic areas of Africa [1]. Hookworm infection due to *Necator americanus* (*Na*) may contribute to poor birth outcomes, malnutrition, poor appetite and anemia especially among children [2] as well as growth retardation and slow cognitive development [3], [4].

Epidemiological studies describing the interaction between helminth and malaria co-infections have shown inconsistent results. Studies suggest a protective effect of helminth infection on *Plasmodium. falciparum* (*Pf*) parasitaemia [5], [6], [7] and disease outcome [8], while others report an increased risk of *Plasmodium* infection [9] and clinical malaria [10], [11] in co-infected individuals. These contradictory observations may be due to differences in host genetics, immune responses, the species of infecting helminth, as well as transmission intensities and exposure [5], [7], [12].

The early stages of *Pf* infection are associated with production of the pro-inflammatory cytokines TNF-α and IFN-γ by T-helper 1 (Th1) cells [13], [14]. In the later stages, there is a switch to Th2 cytokines that stimulate B cells to produce antibodies [7]. The balance between Th1 cytokines (TNF-α, IFN-γ) and Th2 cytokines (IL-10, IL-4) has been shown to be critical in the development of severe falciparum malaria [15] with IL-10 shown to downregulate the functional activity and the production of TNF-α in *Pf* infection [16]. Helminth infection is associated with a Th2 immune response marked by the production of interleukin-4 (IL-4), IL-5 and IL-13 enhancing IgG4 and IgE antibody responses [7] and the expansion of effector cells, including eosinophils, mast cells and basophils [17].

The dynamics involved in the immune responses to *Pf* and *Na* infections are complex and may involve intricate networks of cytokines and other effector mediators which remain poorly understood. Despite the frequent occurrence of these infections and numerous treatment campaigns and control programs [18], [19], studies that have addressed the effect of helminth and malaria coinfections on immune responses in individuals have not directly detailed the specific impact of hookworm concurrent with falciparum malaria [7], [18], [20], [21], [22], [23].

A prior study in Ghana described the cellular cytokine expression in samples collected from subjects living in a multi-parasite endemic area. Lymphocyte subsets stimulated with PHA or LPS were skewed towards an inflammatory phenotype in *Pf* infected samples that was not seen in *Na* infected samples [24]. The study did not compare observations for mono-infected subjects versus co-infected samples and was lacking data assessing the impact of co-infection on the densities of the infecting parasites. The study presented here evaluated stool and blood samples collected from *Na* and *Pf* mono- and co-infected individuals and characterized circulating cytokine plasma levels before and after albendazole treatment.

## 2 Materials and methods

### 2.1 Study site and design

The study was conducted in nine communities located within the Kintampo North Municipality (KNM) in the forest-savannah transitional ecological zone of middle Ghana. The KNM covers a total area of 7162 km^2^ with a population of approximately 140,000 in 32,329 households. The inhabitants are predominantly subsistent farmers of both crop and livestock. The study involved baseline sampling and a follow-up at two weeks post-anthelmintic treatment.

### 2.2 Recruitment of study participants

A durbar was first held in each study village during which the purpose and the nature of the study were explained. A total of 1068 potential study participants aged 4–88 years were randomly identified from a population census data base and recruited into the study. Study subjects (n = 984) who appeared healthy and were without fever were consented individually prior to providing stool and blood samples.

### 2.3 Sample collection and processing

#### 2.3.1 Hookworm

Trained field staff administered a demographic and health questionnaire and provided instruction for the collection of stool in a labeled stool-collection container given to each participant for collection the following day. Fecal samples were collected in a central location and kept cool (25 °C) before microscopic analysis for the presence of helminth eggs using the Kato-Katz method [25], [26]. The intensity of *Na* infections as determined by Kato-Katz method were expressed in eggs per gram (EPG) of feces. Individuals who were positive for hookworm or other soil transmitted nematodes were treated with a single dose of 400 mg albendazole (Remedica, Limassol, Cyprus). Response to treatment was evaluated in stool samples collected 10–14 days post-treatment from all treated subjects.

#### 2.3.2 PCR identification of hookworm species

Hookworm species identification was determined using genomic DNA extracted from purified hookworm eggs [27] samples of infected individuals using QIAamp DNA stool kit (QIAGEN, Hilden, Germany). Purified gDNA (20–40 ng) was used in PCR for the amplification of the internal transcribed region of ribosomal DNA [28]. The PCR reaction contained the forward primer (NC2; 5′-TTA GTT TCT TTT CCT CCG CT-3′), with species specific reverse primers for *A. duodenale* (jmAD; 5′-TGC GAA GTT CGC GTT CGC TGA GC-3′) or *N. americanus* (jmNA; 5′-CGT TAA CAT TGT ATA CCT GTA CAT AC-3′) in separate reactions [28]. The reaction mixtures also contained 1.25 mM each of deoxynucleotide triphosphate (dNTP), 1U of the *Taq* DNA polymerase enzyme (Sigma, Cat. #. D1806-250UN), in reaction buffer. Negative (no template, water) controls were included in all experiments. The PCR cycling conditions were, an initial heating at 94 °C for 5 min, followed by 40 cycles of denaturation at 94 °C for 1 min, annealing at 55 °C for 1 min, and extension at 72 °C for 1 min, with a final elongation step at 72 °C for 5 min. The amplified products were visualized and the sizes determined by UV visualization after electrophoresis in a 2% ethidium bromide stained-agarose gel. Products of the appropriate size (690 bp for *A. duodenale* and 870 bp for *N. americanus*) were considered positive compared to standard controls.

#### 2.3.3 Malaria

Blood collected from individual finger pricks was used to test for asymptomatic malaria with Rapid Diagnostic Test (RDT) kit (CareStart™ Malaria *Pf*HRP2/pLDH Ag RDT, Access Bio, Inc., USA). Thin and thick blood films were prepared and stained with Giemsa prior to examination under the light microscope. Parasite density was estimated against 200 leukocytes in a thick film, assuming a leukocyte count of 8,000 per microliter of blood. Separate samples of blood were captured on Whatman FTA Blood Stain Cards for storage until use in species identification using PCR (see below). All subjects were asymptomatic individuals and were not referred for treatment but cautioned to seek medication at first signs of fever.

### 2.4 PCR identification of *P. falciparum*

Total DNA was extracted from FTA cards using the Chelex method [29]. A 276 bp fragment of *P. falciparum* 18S rRNA gene sequence was amplified using the specific forward 5′-AAC AGA CGG GTA GTC ATG ATT GAG-3′ and reverse 5′-GTA TCT GAT CGT CTT CAC TCCC-3′ primers [30]. The 20 µl reaction contained 20–40 ng total DNA, 0.25 mM of each primer, 1.25 mM of each dNTP, 1U of HotStar *Taq*® DNA polymerase (Biomol GmbH, Hamburg, Germany) and 1X reaction buffer. The PCR conditions were 34 cycles of denaturation at 94 °C for 30 s, annealing at 54 °C for 30 s and extension at 72 °C for 1 min with a final elongation step at 72 °C for 5 min. DNA of the NF54 strain of *P. falciparum* extracted from culture was included on each PCR plate as positive control. The size determination of the amplified PCR products was done as described above.

### 2.5 Cytokine measurements

Approximately 5 ml of whole blood were drawn by venipuncture from each subject. Separation of plasma in EDTA vacutainers tubes was accomplished by centrifugation at 1000*g* for 10 min. Plasma was collected and stored at −80 °C until use in the cytokine assays. Plasma levels of IL-5, IL-10, TNF-α, and CCL11 were determined using the DuoSet ELISA assay reagents (R&D Systems Inc., Minneapolis, USA) following the manufacturer’s instructions.

### 2.6 Statistical analysis

Data analysis was performed using R version 3.3. 2 (https://www.R-project.org/)) software. Proportions such as prevalence were compared between groups (Pearson χ^2^ test). Normalized variables were compared between groups using either the Welch two sample *t* test or One-Way analysis of variance (ANOVA) where appropriate. Pairwise differences between groups were compared using Post Hoc Test (Turkey’s HSD). In assessing the effect of infection status on cytokine levels multivariable logistic regression analysis, were fitted where appropriate. Generalized estimation equation models for panel data with the identity link following the gamma distribution coupled with robust standard errors were fitted to assess if there was any significant difference in cytokine levels before and after albendazole treatment. Parameter estimates and corresponding standard errors were combined using Rubin’s rule [31]. P values < 0.05 were considered statistically significant.

## 3 Results

### 3.1 Demographic and parasitological characteristics of the study population

The overall hookworm prevalence was 10.5% (103/984) while that of *P. falciparum* was 12.4% (122/984). Hookworm and *P falciparum* infected subjects in addition to randomly selected uninfected controls were considered for further evaluation. Of these 198 subjects, 40 were mono-infected with hookworm (*Na*), 59 were infected with *P. falciparum* (*Pf*), and 63 were co-infected with both hookworm and *P. falciparum* (*Na/Pf*). Thirty-six uninfected subjects were randomly selected to serve as uninfected assay controls (n = 36). Other soil transmitted helminths observed by microscopy were either present as mono-infections: *Hymenolepis nana (Hn)* (3.9%)*, Taenia solium (Ts)* (0.8%)*, Trichuris trichiura (Tt)* (1.8%) and *Ascaris lumbricoides (Al)* (0.5%) or co-infections with hookworm (*Na/Hn* = 1.1%; *Na/Ts* = 0.3%; *Na/Tt* = 0.6%; *Na/Al* = 0.3%). Subjects with these helminths, either as mono-infections or co-infections with *Na* were excluded from further analysis. PCR analysis confirmed all hookworm infections were due to only *Na* and none to be *A. duodenale.* Only *Pf* was assessed in PCR, other malaria parasites were not considered.

**Fig. 1:**
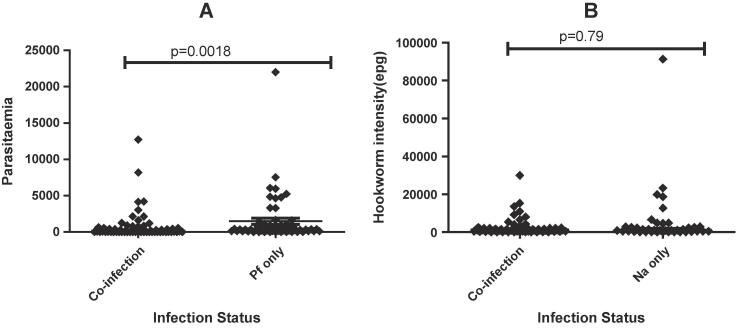
Parasite load among the individual participants. Graph A, shows the geometric mean parasitaemia among individuals with only *P. falciparum* and concurrent *P. falciparum* and *N. americanus* co-infection. Graph B, shows the geometric mean intensity of hookworm mono-infections and co-infection with *P. falciparum*. epg; egg per gram of stool, *Na; Necator americanus*. Pf; *Plasmodium falciparum.*

A significant difference was found between gender and infection status [χ^2^_(3, 198)_ = 15.7, p = 0.0013]. There was no significant difference (p = 0.79) between the geometric mean intensity of hookworm mono-infections (1182.0 EPG; 95%CI [704.9, 1981.9]) and the EPG for co-infected groups (1087.5 EPG; 95% CI [782.7, 1511.0]). The geometric mean *P. falciparum* parasitaemia was significantly higher in *Pf* mono-infected subjects (338.7/µl of blood; 95% CI [210.1, 546.0]) than in co-infected subjects (109.4/µl of blood; 95% CI [66.1, 181.0]) [Welch Two Sample t _(120)_ = −3.2, p = 0.0018] (Fig. 1).

### 3.2 Association between parasite infection status and cytokine levels

The analysis of variance results showed that there was statistically significant difference (p < 0.05) in the mean cytokine levels among disease classification (negative controls, hookworm only, *P. falciparum* only, hookworm and *P. falciparum* co-infection) (Table 1a). Pairwise mean differences of the cytokine levels between all the infected groups showed that individuals infected with only *Na* had significantly higher CCL11 and IL-5 levels compared to those with either *Pf* only or concurrent *Na/Pf* infections. IL-10 level was significantly higher in the co-infected individuals compared to the single infected or the uninfected group. TNF-α levels were higher in all infected groups compared to the uninfected controls (Table 1a).

**Table 1:** Association between parasite and cytokine levels.

aMean cytokine level (pg/ml) among the infection status [Raw Data]								
	CCL11±sem	p-value	IL-10±sem	p-value	IL-5±sem	p-value	TNF-α ± sem	p-value
Uninfected	45.9 ± 3.9^b^	<0.001	10.7 ± 7.6^b^	<0.001	4.3 ± 2.7^b^	0.027	3.4 ± 2.1^a^	0.0038
*N. americanus* only	98.5 ± 19.2^a^		14.9 ± 3.3^ab^		10.5 ± 4.1^a^		5.9 ± 1.7^b^	
*P. falciparum* only	34.8 ± 5.2^b^		18.9 ± 3.1^ab^		3.8 ± 1.8^b^		5.6 ± 1.2^b^	
*Na* + *Pf* co-infected	78.5 ± 12.4^a^		39.9 ± 12.2^a^		2.8 ± 1.0^b^		7.3 ± 1.2^b^	

bAssessing the effect of disease classification status on the cytokine levels [log_10_ transformed data]								
Covariate	Log CCL11 β[95%CI]	p-value	Log IL-10 β[95%CI]	p-value	Log IL-5 β[95%CI]	p-value	Log TNF-α β[95%CI]	p-value
Age in years	0.01 [−0.00, 0.01]	0.092	−0.04 [−0.07, −0.02]	0.001	−0.01 [−0.03, 0.01]	0.32	−0.03 [−0.05, −0.02]	0.00065
Male	ref		ref		ref		ref	
Females	0.13 [−0.08, 0.34]	0.220	−0.14 [−0.88, 0.59]	0.700	−0.18 [−0.87, 0.52]	0.62	−0.65 [−1.26, −0.03]	0.042
Uninfected	ref		ref		ref		ref	
*N. americanus* only	0.47 [0.15, 0.80]	0.005	2.40 [1.26, 3.54]	<0.0001	0.98 [−0.09, 2.05]	0.075	1.27 [0.32, 2.23]	0.01
*P. falciparum* only	−0.24 [−0.57, 0.10]	0.170	2.11 [0.94, 3.28]	0.001	−0.11 [−1.21, 1.00]	0.85	1.26 [0.27, 2.24]	0.014
*Na* + *Pf* co-infected	0.45 [0.13, 0.78]	0.007	2.08 [0.95, 3.22]	0.001	0.03 [−1.04, 1.10]	0.96	0.59 [−0.37, 1.54]	0.23

a Univariate analysis for estimating difference in cytokine levels by disease classification using one way analysis of variance. The p value is by ANOVA. Superscripts with different letters are significant difference [Turkey’s contrast].

b Multivariate multiple linear regression analysis adjusting for age and sex. β: Estimated effect of covariate on cytokine level, CI: Confidence interval, Na: *Na, Necator americanus*; *Pf, Plasmodium falciparum.*

In a multiple linear regression analysis adjusting for age and gender with the uninfected group as the reference, CCL11 levels were significantly higher in subjects infected with *Na* only (β = 0.47, 95%CI [0.15, 0.80], p = 0.005) or co-infected with *Pf* (β = 0.45, 95%CI [0.13, 0.78], p = 0.007). IL-10 levels were higher in all infected groups [*Na* only: (β = 2.40, 95%CI [1.26, 3.54], p < 0.0001); *Pf* only: (β = 2.11, 95% CI [0.94–3.28], p = 0.0005) and *Na/Pf* co-infected: (β = 2.08, 95%CI [0.95, 3.22], p = 0.00043)] compared to the uninfected controls (Table 1b). In contrast, there was no significant difference (p > 0.05) in IL-5 levels between any of the infected groups and the uninfected control. TNF-α levels were higher only for the *Na* and *Pf* mono-infection groups (Table 1b).

### 3.3 Association between *N. americanus* infection intensity and cytokine levels

The relationship between cytokine levels and the intensity of *Na* infection (EPG) was assessed by linear regression analysis and Spearman’s correlation. There was a significantly negative correlation (r_s_ = −0.39, n = 40, p = 0.021) between *Na* intensity and CCL11 levels in the group infected with only *Na*. In contrast, although insignificant, there was a trend of positive correlation (r_s_ = 0.11, n = 40, p = 0.45) between *Na* infection intensity and CCL11 levels in the group with *Na/Pf* co-infection. No other significant correlations were noted with *Na* infection intensity.

### 3.4 Effect of albendazole treatment on parasite intensity and cytokine levels

**Fig. 2:**
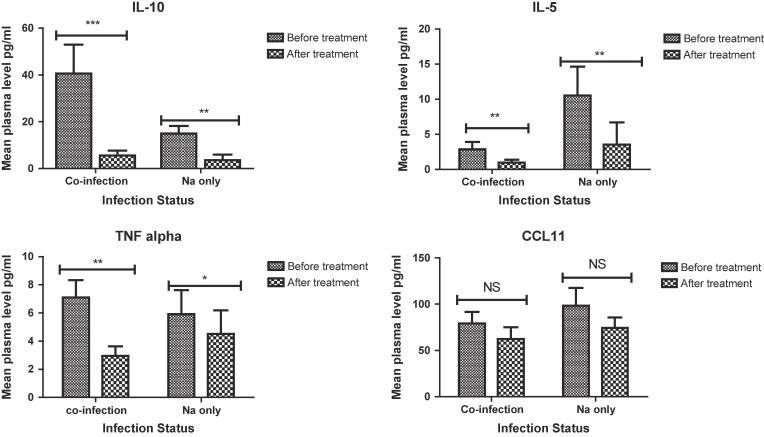
Mean plasma cytokine levels among hookworm infected individual before and after albendazole treatment. Mean plasma levels are shown for pairwise comparisons of the various infected groups (mono- and co-infected). Pairwise differences that are statistically significant are indicated by asterisks. Significant levels; ***p < 0.001, **p < 0.01, *p < 0.05, ^NS^p > 0.05. Error bar represent standard error of the mean. Abbreviations: Na: *Necator americanus* (40); Co-infection: hookworm and *P. falciparum* co-infected, Sixty three (n = 63) coinfected and 40 hookworm only infected individuals were recruited for pre- treatment assay. Eighty seven (87) individuals who were successfully treated (no egg present in stool) after albendazole administration blood samples were collected for the post treatment cytokine assay. Of these 51 were coinfected and 35 were only infected with hookworm. Blood sample were not collected from the 16 treatment failure individuals.

The overall cure rate of albendazole treatment was 84.5% (87/103) based on 10–14 days post-treatment stool examinations. Treatment with albendazole led to a significant reduction of TNF-α (β = −0.75; 95% CI [−1.47, −0.03]; p = 0.041) and IL-5 (β = −0.91; 95% CI [−1.60, −0.22]; p = 0.01) levels. IL-10 also decreased following deworming treatment by (β = −2.98; 95% CI [−3.68, −2.27]; p = 0.001) as shown by generalized fixed effect model (Table 2). There was no significant change in CCL11 levels after albendazole treatment (Table 2). A pairwise comparison of the mean cytokine levels between all the infected groups (mono and co-infection) before and after albendazole treatment showed that IL-10, IL-5 and TNF-α levels are reduced after treatment in both individuals infected with only hookworm or concurrent hookworm and *P. falciparum* infections (Fig. 1). However, there was no significant difference in the mean CCL11 level among hookworm only or coinfected groups before and after treatment, although there was slight reduction (Fig. 2).

**Table 2:** Treatment effect of albendazole on cytokine levels.

Cytokine	Pre (mean ± sem)	Post (mean ± sem)	β	95%CI	p-value
CCL11	3.85 ± 0.06	3.96 ± 0.08	−0.07	−0.18, 0.04	0.19
IL-10	1.46 ± 0.20	−0.93 ± 0.33	−2.98	−3.68, −2.27	0.001
IL-5	−0.97 ± 0.17	−1.68 ± 0.24	−0.91	−1.60, −0.22	0.01
TNF-α	0.33 ± 0.16	−0.10 ± 0.28	−0.75	−1.47, −0.03	0.041

Generalized Estimating Equation was used to determine the effect of albendazole treatment on cytokine levels (log transformed).β: Effect of albendazole treatment on cytokine levels, CI: confidence interval. Reference category-before treatment with albendazole, sem: standard error of the mean.

## 4 Discussion

The immunological outcomes of the interactions between hookworm and malaria infections may be important in understanding not only their associated pathophysiology and morbidity but could also be useful in developing interventions against these parasites. In this study, the relationship between hookworm and malaria (either single or co-infections) and their infection intensity as well as host cytokine responses were assessed. The intensity of *Na* infection was not different between individuals infected with *Na* only and those with concurrent *Pf* infection. In contrast, *Pf* parasite density was significantly lower in the co-infected group than in those singly infected with only *Pf* suggesting that *Na* may contribute to reducing malaria parasitaemia. By comparison, previous cross sectional studies conducted in Tanzania and Ethiopia reported a significant positive correlation between *Pf* density and *Na* infection intensity [32], [33] while Salim et al. [34] found no such association. The interactions between *Na* and malaria may be influenced by multiple factors, including transmission intensity, immunological and nutritional status as well as age of the infected person [35], [36], [37]. For instance, our study found a negative association between *Na* infection status and malaria parasitaemia in children less than 15 years old but not in those who were older, However, a previous study by Humphries et al. [1], showed that school-age children with hookworm infection were nearly three times more likely to have a positive malaria smear than those who were hookworm-negative. Our study also showed that children coinfected with *Na* and *Pf* were less likely to be anemic than those singly infected with *Pf*, suggesting that hookworm conferred partial protection against malarial anemia [1].

TNF-α is involved in the induction of fever, which may contribute to suppressing malaria parasitaemia [38], [39]. Although the study participants were afebrile, the higher TNF-α levels observed in individuals singly infected with *Na* or *Pf* compared to the uninfected individuals may be an indication of an ongoing parasite-mediated inflammatory response [40]. However, other anti-inflammatory mediators such as IL-10 are important in keeping TNF-α levels in balance in order to minimize potential adverse effects of prolonged elevated levels of TNF-α to the host [39]. This may explain why the level of IL-10 was higher in the *Na* or *Pf* infected individuals and especially very high in the co-infected individuals. However, the negative correlation reported between Th2 cytokine levels and worm burden in other helminthic infections such as *Ascaris lumbricoides* [41], were not observed in this hookworm infected cohort. While *Na* and *Pf* co-infected subjects had TNF-α levels comparable to those who were uninfected their IL-10 levels were higher. This may be indicative of a stronger immunomodulatory effect of *Na* in co-infected individuals, thus driving the immune response towards a more immune-tolerant state favourable for its survival.

Eotaxin acts systemically, together with IL-5, to stimulate the release of eosinophils from the bone marrow, and locally to mediate their selective recruitment to sites of inflammation [42], [43]. Eosinophils may degranulate to release their inflammatory mediators and molecules such as eosinophil cationic protein, killing or aiding in the expulsion of invading helminths from the body [43]. This may account for the significant higher CCL11 and IL-5 levels in the hookworm infected individuals. This further, may account for the significant negative correlation between *Na* infection intensity and CCL11 levels in the group infected with hookworm only. The higher level of CCL11 found in individuals with *Na* infections corroborates the idea that the production of enzymes inactivating eotaxin may be a strategy employed by helminths to prevent recruitment and activation of eosinophils at the site of infection [44]. Although in the *Na/Pf* co-infected group, CCL11 levels were higher compared to the uninfected group, it is not clear why there was no correlation with worm intensity. Perhaps the presence of *Pf* together with *Na* may trigger other cytokines such as IFN–γ that could direct other downstream effectors of CCL11 functionality away from hookworm in the co-infected individuals. For instance, CCL11 mediated eosinophilia which may be key in host defense against *Na* is also a hallmark of *Pf* infection [45]. It is therefore possible that in the co-infected individuals the overall potency of this mechanism against any particular parasite is limited by their independent modulations of the immune system. However, higher CCL11 levels was observed in both the *Na* only and the co-infected groups compared to the *Pf* only and the uninfected groups, suggesting that the presence of *Na* may have contributed significantly to the increased levels of CCL11. By contrast, none of the other cytokines investigated showed any significant correlation with *Na* load, which precludes an assessment of their specific role modulating hookworm infection intensity.

We observed a significant decline in TNF-α, IL-5 and IL-10 levels after albendazole treatment in the *Na* infected individuals (*Na* only and co-infected group). It is possible that deworming alleviates the immune pressure responsible for the increased cytokine levels, thus causing a return to the resting state. This observation is contrast to other studies, which found a boosting of Th2 cytokine responses following praziquantel treatment against schistosomiasis [46], [47]. The differences in cytokine response for the two different helminth after treatment may be attributed to the different sites of infection of these two helminths (hookworms and schistosomes) and modes of action of the anthelmintic drugs (albendazole and praziquantel). *Na* inhabits the small intestine, while schistosomes reside in the mesenteric veins. Whereas praziquantel causes disruption of the tegument and intravascular exposure to antigen, albendazole causes metabolic disruption, resulting in the paralysis and death of worms, which are then expelled intact from the gut within 2 weeks [48]. Thus, with effective clearance of intestinal hookworms following albendazole treatment, host cytokine levels likely return to their baseline, “unstimulated” state.

## 5 Conclusion

This study has demonstrated that *Na* and *Pf* parasite co-infection exhibits altered human cytokine profiles, and that coinfection may be associated with reduced malaria parasitaemia. Further comprehensive studies that include characterization of host cellular immune responses are needed to fully define the unique effects of these two globally important parasitic diseases.

## 6 Ethics statement

The study protocol was reviewed and approved by the Institutional Review Board of Noguchi Memorial Institute for Medical Research (FWA#: 00001824) prior to the initiation of the project. An information session was presented to the study participants prior to obtaining written consent. Adolescents and children provided assent in addition to the required informed consent of a parent or guardian.

## Funding

This work was supported by 10.13039/100000002National Institutes of Health, grant ID #: 1R01AI099623 award MDW. The funders had no role in study design, data collection and analysis, decision to publish, or preparation of the manuscript.

## Authors contribution

BA, BA, MTF, BG and MDW designed, and play major contribution in performing and writing the manuscript. BA, BA, and DD performed the statistical analyses. BA, MDW and TSA were responsible for patient recruitment, parasitological and molecular examination. BA performed immunological assay. SVN, EKA, LMH, MC contribute in writing the paper. MDW, and MC provided resources for the study. BA, BG, MTF and MDW supervise the study. All authors read and approved the final manuscript.

## Declaration of Competing Interest

The authors declare that they have no known competing financial interests or personal relationships that could have appeared to influence the work reported in this paper.

## References

[1] Humphries D., Mosites E., Otchere J., Twum W.A., Woo L., Jones-Sanpei H. (2011). Epidemiology of hookworm infection in Kintampo North Municipality, Ghana: patterns of malaria coinfection, anemia, and albendazole treatment failure. Am. J. Trop. Med. Hygiene.

[2] Ndyomugyenyi R., Kabatereine N., Olsen A., Magnussen P. (2008). Malaria and hookworm infections in relation to haemoglobin and serum ferritin levels in pregnancy in Masindi district, western Uganda. Trans. R. Soc. Trop. Med. Hyg..

[3] Dickson R., Awasthi S., Williamson P., Demellweek C., Garner P. (2000). Effects of treatment for intestinal helminth infection on growth and cognitive performance in children: systematic review of randomised trials. BMJ.

[4] Stoltzfus R.J., Kvalsvig J.D., Chwaya H.M., Montresor A., Albonico M., Tielsch J.M. (2001). Effects of iron supplementation and anthelmintic treatment on motor and language development of preschool children in Zanzibar: double blind, placebo controlled study. BMJ.

[5] Lyke K.E., Dicko A., Dabo A., Sangare L., Kone A., Coulibaly D. (2005). Association of *Schistosoma haematobium* infection with protection against acute Plasmodium falciparum malaria in Malian children. Am. J. Trop. Med. Hygiene.

[6] Brutus L., Watier L., Hanitrasoamampionona V., Razanatsoarilala H., Cot M. (2007). Confirmation of the protective effect of *Ascaris lumbricoides* on *Plasmodium falciparum* infection: results of a randomized trial in Madagascar. Am. J. Trop. Med. Hygiene.

[7] Courtin D., Djilali-Saïah A., Milet J., Soulard V., Gaye O., Migot-Nabias F. (2011). *Schistosoma haematobium* infection affects *Plasmodium falciparum*-specific IgG responses associated with protection against malaria. Parasite Immunol..

[8] Nacher M., Gay F., Singhasivanon P., Krudsood S., Treeprasertsuk S., Mazier D. (2000). Ascaris lumbricoides infection is associated with protection from cerebral malaria. Parasite Immunol..

[9] Midzi N., Sangweme D., Zinyowera S., Mapingure M., Brouwer K., Munatsi A. (2008). The burden of polyparasitism among primary schoolchildren in rural and farming areas in Zimbabwe. Trans. R. Soc. Trop. Med. Hyg..

[10] Nacher M., Singhasivanon P., Yimsamran S., Manibunyong W., Thanyavanich N., Wuthisen P. (2002). Intestinal helminth infections are associated with increased incidence of *Plasmodium falciparum* malaria in Thailand. J. Parasitol..

[11] Spiegel A., Tall A., Raphenon G., Trape J.F., Druilhe P. (2003). Increased frequency of malaria attacks in subjects co-infected by intestinal worms and *Plasmodium falciparum* malaria. Trans. R. Soc. Trop. Med. Hyg..

[12] Boef A., May L., Van Bodegom D., Kuningas M., Eriksson U., Westendorp R. (2012). The influence of genetic variation on innate immune activation in an environment with high infectious pressure. Genes Immun..

[13] Clark I., Cowden W., Butcher G., Hunt N. (1987). Possible roles of tumor necrosis factor in the pathology of malaria. Am. J. Pathol..

[14] Lyke K., Burges R., Cissoko Y., Sangare L., Dao M., Diarra I. (2004). Serum levels of the proinflammatory cytokines interleukin-1 beta (IL-1β), IL-6, IL-8, IL-10, tumor necrosis factor alpha, and IL-12 (p70) in Malian children with severe *Plasmodium falciparum* malaria and matched uncomplicated malaria or healthy controls. Infect. Immun..

[15] Othoro C., Lal A.A., Nahlen B., Koech D., Orago A.S., Udhayakumar V. (1999). A low interleukin-10 tumor necrosis factor-α ratio is associated with malaria anemia in children residing in a holoendemic malaria region in western Kenya. J. Infect. Dis..

[16] Wenisch C., Parschalk B., Narzt E., Looareesuwan S., Graninger W. (1995). Elevated serum levels of IL-10 and IFN-γ in patients with acute *Plasmodium falciparum* malaria. Clin. Immunol. Immunopathol..

[17] R.M. Maizels, D.A. Bundy, M.E. Selkirk, D.F. Smith, R.M. Anderson, Immunological modulation and evasion by helminth parasites in human populations, 1993. 10.1038/365797a0 8413664

[18] Hamm D.M., Agossou A., Gantin R.G., Kocherscheidt L., Banla M., Dietz K. (2009). Coinfections with *Schistosoma haematobium*, *Necator americanus* and *Entamoeba histolytica/Entamoeba dispar* in Children: chemokine and cytokine responses and changes after antiparasite treatment. J. Infect. Dis..

[19] Kabatereine N.B., Brooker S., Koukounari A., Kazibwe F., Tukahebwa E.M., Fleming F.M. (2007). Impact of a national helminth control programme on infection and morbidity in Ugandan schoolchildren. Bull. World Health Organ..

[20] Maizels R.M., Balic A., Gomez-Escobar N., Nair M., Taylor M.D., Allen J.E. (2004). Helminth parasites–masters of regulation. Immunol. Rev..

[21] Nacher M. (2004). Interactions between worm infections and malaria. Clin. Rev. Allergy Immunol..

[22] Hartgers F., Yazdanbakhsh M. (2006). Co-infection of helminths and malaria: modulation of the immune responses to malaria. Parasite Immunol..

[23] Reilly L., Magkrioti C., Mduluza T., Cavanagh D., Mutapi F. (2008). Effect of treating *Schistosoma haematobium* infection on *Plasmodium falciparum*-specific antibody responses. BMC Infect. Dis..

[24] Boef A.G., May L., van Bodegom D., van Lieshout L., Verweij J.J., Maier A.B. (2013). Parasitic infections and immune function: effect of helminth infections in a malaria endemic area. Immunobiology.

[25] John D.T., Petri W.A., Markell E.K., Voge M. (2006). Markell and Voge's Medical Parasitology.

[26] WHO (1998). Guidelines for the Evaluation of Soil-Transmitted Helminthiasis and Schistosomiasis at Community Level.

[27] Humphries D., Simms B.T., Davey D., Otchere J., Quagraine J., Terryah S. (2013). Hookworm infection among school age children in Kintampo North Municipality, Ghana: nutritional risk factors and response to albendazole treatment. Am. J. Trop. Med. Hygiene.

[28] Monti J., Chilton N., Bao-Zhen Q., Gasser R. (1998). Specific amplification ofNecator americanusorAncylostoma duodenaleDNA by PCR using markers in ITS-1 rDNA, and its implications. Mol. Cell. Probes.

[29] Snounou G. (2002). Genotyping of *Plasmodium* spp. nested PCR. Malaria Methods Protocols: Methods Protocols.

[30] Padley D., Moody A., Chiodini P., Saldanha J. (2003). Use of a rapid, single-round, multiplex PCR to detect malarial parasites and identify the species present. Ann. Trop. Med. Parasitol..

[31] Rubin D.B. (1996). Multiple imputation after 18+ years. J. Am. Stat. Assoc..

[32] Degarege A., Animut A., Legesse M., Erko B. (2009). Malaria severity status in patients with soil-transmitted helminth infections. Acta Trop..

[33] Mboera L.E., Senkoro K.P., Rumisha S.F., Mayala B.K., Shayo E.H., Mlozi M.R. (2011). Plasmodium falciparum and helminth coinfections among schoolchildren in relation to agro-ecosystems in Mvomero District, Tanzania. Acta Tropica.

[34] Salim N., Knopp S., Lweno O., Abdul U., Mohamed A., Schindler T. (2015). Distribution and risk factors for *Plasmodium* and helminth co-infections: a cross-sectional survey among children in Bagamoyo district, Coastal Region of Tanzania. PLoS Negl. Trop. Dis..

[35] Pullan R.L., Kabatereine N.B., Bukirwa H., Staedke S.G., Brooker S. (2011). Heterogeneities and consequences of *Plasmodium* species and hookworm coinfection: a population based study in Uganda. J. Infect. Dis..

[36] Yatich N.J., Yi J., Agbenyega T., Turpin A., Rayner J.C., Stiles J.K. (2009). Malaria and intestinal helminth co-infection among pregnant women in Ghana: prevalence and risk factors. Am. J. Trop. Med. Hygiene.

[37] Kinung'hi S.M., Magnussen P., Kaatano G.M., Kishamawe C., Vennervald B.J. (2014). Malaria and helminth co-infections in school and preschool children: a cross-sectional study in Magu district, north-western Tanzania. PLoS ONE.

[38] Nacher M., Singhasivanon P., Traore B., Dejvorakul S., Phumratanaprapin W., Looareesuwan S. (2001). Hookworm infection is associated with decreased body temperature during mild *Plasmodium falciparum* malaria. Am. J. Trop. Med. Hygiene.

[39] Othoro C., Lal A.A., Nahlen B., Koech D., Orago A.S., Udhayakumar V. (2017). A low interleukin-10 tumor necrosis factor-α ratio is associated with malaria anemia in children residing in a holoendemic malaria region in western Kenya. J. Infect. Dis..

[40] Geiger S., Massara C., Bethony J., Soboslay P., Correa-Oliveira R. (2004). Cellular responses and cytokine production in post-treatment hookworm patients from an endemic area in Brazil. Clin. Exp. Immunol..

[41] Turner J.D., Faulkner H., Kamgno J., Cormont F., Van Snick J., Else K.J. (2003). Th2 cytokines are associated with reduced worm burdens in a human intestinal helminth infection. J. Infect. Dis..

[42] Palframan R.T., Collins P.D., Williams T.J., Rankin S.M. (1998). Eotaxin induces a rapid release of eosinophils and their progenitors from the bone marrow. Blood.

[43] Parham (2004). The Immune System.

[44] Culley F.J., Brown A., Conroy D.M., Sabroe I., Pritchard D.I., Williams T.J. (2000). Eotaxin is specifically cleaved by hookworm metalloproteases preventing its action in vitro and in vivo. J. Immunol..

[45] Kurtzhals J., Reimert C., Tette E., Dunyo S., Koram K., Akanmori B. (1998). Increased eosinophil activity in acute *Plasmodium falciparum* infection-association with cerebral malaria. Clin. Exp. Immunol..

[46] Ho M., Sexton M.M., Tongtawe P., Looareesuwan S., Suntharasamai P., Webster H.K. (1995). Interleukin-10 inhibits tumor necrosis factor production but not antigen-specific lymphoproliferation in acute *Plasmodium falciparum* malaria. J. Infect. Dis..

[47] Scott J.T., Mutapi F., Woolhouse M.E., Chandiwana S.K., Mduluza T., Ndhlovu P.D. (2000). Dissociation of interleukin-4 and interleukin-5 production following treatment for *Schistosoma haematobium* infection in humans. Parasite Immunol..

[48] Keiser J., Utzinger J. (2008). Efficacy of current drugs against soil-transmitted helminth infections: systematic review and meta-analysis. JAMA.

